# Personalized, home-based transcranial alternating current stimulation for memory improvement in mild cognitive impairment: randomized, sham-controlled clinical trial protocol

**DOI:** 10.3389/fnagi.2026.1882406

**Published:** 2026-07-31

**Authors:** Lucie Bréchet, Camille Bouhour, Vincent Rochas, Valérie A. Mekrom, Mohamed Eshmawey, Paul G. Unschuld

**Affiliations:** 1 Department of Clinical Neurosciences, University of Geneva, Geneva, Switzerland; 2 Foundation Campus Biotech, Geneva, Switzerland; 3 Geriatric Psychiatry Service, University Hospitals of Geneva (HUG), Thônex, Switzerland; 4 Department of Psychiatry, University of Geneva, Geneva, Switzerland

**Keywords:** electroencephalography, gamma oscillations, mild cognitive impairment, personalized neuromodulation, randomized controlled trial, transcranial alternating current stimulation

## Abstract

**Background:**

Mild cognitive impairment (MCI) is a prodromal stage of Alzheimer’s disease (AD) and represents a critical window for interventions aimed at slowing neurodegenerative progression. Disruptions in neural oscillatory activity are key contributors to memory dysfunction in MCI and AD. Transcranial alternating current stimulation (tACS) is a non-invasive neuromodulation technique that entrains neural oscillations at specific frequencies, potentially restoring network dynamics underlying memory.

**Methods:**

The MemStim study is a randomized, sham-controlled, parallel-arm, double-blind clinical trial evaluating the efficacy of personalized, home-based gamma-frequency tACS over the left angular gyrus, delivered 5 days a week for 4 weeks, in individuals with MCI. Safety and tolerability are monitored throughout the intervention. Forty participants aged ≥55 years with clinically diagnosed MCI will be recruited at the Geneva University Hospitals and randomized to active or sham stimulation. All participants undergo baseline and post-stimulation assessments, including cognitive testing, structural MRI, and high-density EEG (hdEEG). Individual MRI-based electric-field modeling personalizes stimulation montages targeting the left angular gyrus, a key node of the memory network.

**Outcomes:**

The primary outcome is the change in global cognitive function measured by the Montreal Cognitive Assessment (MoCA) from baseline to post-intervention. Secondary outcomes include changes in gamma-band oscillatory power measured with hdEEG. Exploratory EEG analyses will examine spectral, temporal, and spatial brain dynamics. Cognitive assessments are repeated 3 months post-intervention to evaluate persistence of effects.

**Discussion:**

This trial investigates whether personalized gamma-frequency tACS can modulate hdEEG measures of resting-state brain activity and improve memory function in MCI. By integrating individualized stimulation targeting, electrophysiological biomarkers, and remotely supervised home-based delivery, the MemStim study aims to advance personalized neuromodulation as a scalable therapeutic strategy for early intervention in cognitive decline.

**Clinical trial registration:**

ClinicalTrials.gov, identifier NCT05708001.

## 1 Background

Cognitive decline in Alzheimer’s disease (AD) is increasingly recognized to involve disruptions of large-scale brain network dynamics in addition to structural neurodegeneration. Mild cognitive impairment (MCI), widely considered a prodromal stage of AD, provides a critical window to investigate interventions targeting early disease processes. Individuals with MCI are at elevated risk of developing dementia, with reported annual conversion rates estimated between 10 and 30% (Petersen, 2004; Mitchell and Shiri-Feshki, 2009). Given the gradual nature of AD and the limited effectiveness of currently available pharmacological treatments in reversing cognitive decline, early intervention during the MCI stage represents a priority for strategies aimed at slowing or preventing disease progression (Cummings et al., 2022).

These network disruptions predominantly involve alterations in brain oscillations, i.e., rhythmic fluctuations of neuronal activity that coordinate communication across distributed brain regions, which have been identified as key mechanisms underlying cognitive dysfunction (Buzsaki and Draguhn, 2004; Anticevic et al., 2012). Brain oscillations emerge from coordinated neuronal interactions within and between brain regions that can be measured non-invasively using electroencephalography (EEG). By synchronizing neuronal firing across distributed neural networks, oscillatory activity plays a fundamental role in neural communication, synaptic plasticity, and cognitive processing (Moretti et al., 2017; Vosskuhl et al., 2018).

Among the different frequency bands, gamma oscillations (typically 30–80 Hz) are particularly relevant for higher cognitive functions, including attention, working memory, and episodic memory (Buzsaki and Draguhn, 2004; Herrmann and Demiralp, 2005). Gamma activity is prominent within hippocampal-cortical networks involved in memory encoding and retrieval, and is thought to support the integration of distributed neural representations (De Paolis et al., 2024). Disruptions in gamma oscillations have been reported in individuals with MCI and AD and are associated with synaptic dysfunction and impaired network synchronization (Moretti et al., 2017). These findings suggest that restoring or modulating gamma-oscillatory activity may be a promising strategy to improve cognitive function in individuals with MCI and AD (De Paolis et al., 2024).

Non-invasive brain stimulation techniques have emerged as promising tools to modulate neural oscillations associated with cognitive processes. Among these approaches, transcranial alternating current stimulation (tACS) is particularly well suited to interact with endogenous brain rhythms (Wischnewski et al., 2023). tACS delivers weak sinusoidal electrical currents at a specific frequency through scalp electrodes, generating oscillatory electric fields that can influence neuronal membrane potentials and entrain ongoing neural oscillations (Antal et al., 2017; Agboada et al., 2025). Because stimulation frequency can be precisely controlled, tACS offers a promising approach for modulating pathological oscillatory activity (Vosskuhl et al., 2018).

Early clinical studies have provided encouraging evidence supporting the therapeutic potential of gamma-frequency tACS in AD and MCI, as summarized in recent reviews of non-invasive brain stimulation in AD (De Paolis et al., 2024; Nissim et al., 2023; Manippa et al., 2024; Koch et al., 2024). In a randomized sham-controlled, lab-based study, Benussi et al. (2022) demonstrated that gamma-frequency tACS targeting the precuneus significantly improved episodic memory performance and restored measures of cholinergic transmission in patients with early AD, providing evidence that modulation of gamma oscillations may influence cognitive function in neurodegenerative disorders.

A critical consideration for clinical translation is the role of anatomical variability. Particularly in aging and neurodegenerative populations, interindividual differences in head anatomy can substantially influence the distribution and intensity of electric fields generated by tACS. Advances in computational modeling now allow the use of structural magnetic resonance imaging (MRI) to generate individualized head models and optimize electrode configurations, thereby enabling more precise targeting of specific brain networks (Thielscher et al., 2015; Ruffini et al., 2014; Miranda et al., 2013). These methodological advances have informed the development of personalized tACS protocols in which the current distribution across a multi-electrode configuration is optimized for each individual based on their specific neuroanatomy and tissue conductivities.

In this context, we have previously proposed that personalized gamma-frequency tACS targeting the angular gyrus network may help reduce episodic memory deficits in AD (Bréchet et al., 2021a). In a methodological analysis conducted within the framework of the present trial, we demonstrated that MRI-individualized multi-electrode tACS significantly enhances the induced electric field strength in the targeted region in patients with MCI compared with standardized stimulation approaches (Rochas et al., 2025), underscoring the importance of personalized modeling in this population.

This line of research was initially explored in a proof-of-concept study conducted at Harvard Medical School, in which a patient-tailored, caregiver-administered home-based gamma-frequency tACS protocol targeting memory-related networks was developed and shown to be feasible and safe, with two patients successfully completing 70 sessions over 14 weeks under remote supervision (Bréchet et al., 2021b). Building on this initial work, subsequent studies have further explored home-based transcranial electrical stimulation in AD, including a randomized clinical trial demonstrating that repeated home-based gamma-frequency tACS is feasible and may improve episodic memory performance (Cantoni et al., 2025), as well as additional proposed protocols and trials (Cappon et al., 2023; Tang et al., 2024; Altomare et al., 2023). In a parallel qualitative analysis conducted within the present trial, we demonstrated that family caregivers can reliably administer repeated tACS sessions under remote supervision, reporting high levels of confidence in device operation and overall acceptability of the intervention (Bouhour et al., 2026). Together, these efforts suggest that home-based neuromodulation is feasible in AD, yet evidence in MCI remains scarce, and few studies have combined home-based stimulation with individualized, anatomy-based targeting. Intervening at the MCI stage may therefore provide an opportunity to modulate dysfunctional neural network dynamics before widespread neurodegeneration and irreversible network disruption occur.

To our knowledge, this represents one of the first randomized clinical trials to combine individualized MRI-guided targeting with electrophysiological biomarkers to evaluate gamma-frequency tACS in MCI. By integrating individualized stimulation targeting, electrophysiological biomarkers, and remotely supervised home-based delivery, the MemStim study aims to determine whether modulation of gamma oscillations can improve memory function in individuals with MCI at risk for AD.

## 2 Objectives

The overall objective of the MemStim study is to examine the efficacy of remote, caregiver-administered gamma-frequency transcranial alternating current stimulation on cognitive function in individuals with MCI. The primary objective is to evaluate whether personalized gamma-frequency transcranial alternating current stimulation improves global cognitive function, as measured by the Montreal Cognitive Assessment (MoCA). Global cognition was chosen as the primary endpoint for several reasons: it is the pre-specified, registered outcome; gamma-frequency tACS in neurodegenerative disease has been associated with broad rather than exclusively episodic cognitive benefits; and a clinically and regulatory-relevant global endpoint is appropriate for a scalable intervention and permits benchmarking against prior gamma-tACS trials. Mechanism-linked memory measures, including a comprehensive episodic memory battery, working memory measures, the MoCA Memory Index Score, and behavioral performance on the autobiographical memory task, are captured as prespecified exploratory outcomes and provide a more direct behavioral test of the proposed mechanism, complementing the source-level gamma-band response recorded during the same task. The secondary objective is to assess changes in gamma-band oscillatory activity before and after the home-based stimulation intervention using high-density EEG (hdEEG). Safety and tolerability of the Starstim-Home tES device are monitored as a prespecified safety outcome, with systematic adverse-event reporting in accordance with ISO 14155.

## 3 Methods

### 3.1 Study design

**Figure 1:**
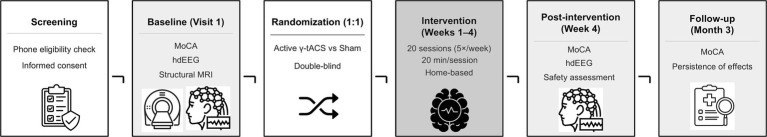
Study timeline of the MemStim randomized controlled trial. Participants undergo screening and baseline assessments, including cognitive testing, high-density electroencephalography (hdEEG), and structural MRI. After randomization (1:1), participants receive either active gamma-frequency transcranial alternating current stimulation (*γ*-tACS) or sham stimulation during 20 home-based sessions over 4 weeks. Post-intervention assessments are conducted after completion of the stimulation period, followed by a three-month follow-up visit to evaluate persistence of effects.

Following a telephone screening and written informed consent, participants undergo baseline assessments, including cognitive testing using the Montreal Cognitive Assessment (MoCA), high-density EEG (hdEEG), and structural MRI. Participants are then randomly assigned 1:1 to receive either active gamma-frequency tACS (*γ*-tACS) or sham stimulation. The intervention consists of 20 home-based sessions over 4 weeks (5 sessions per week, 20 min per session), administered by a caregiver or trained study staff member under remote supervision. Post-intervention assessments include MoCA, hdEEG, and safety evaluation. A follow-up visit at 3 months after the end of stimulation assesses the persistence of potential effects using MoCA (Figure 1).

### 3.2 Participants

Participants with MCI are recruited through the Geriatric Psychiatry Service and the Memory Clinic of the Geneva University Hospitals (HUG), as well as through community advertisements and existing research registries. All study procedures are conducted at Campus Biotech in Geneva. Potential participants first undergo a phone screening to determine preliminary eligibility. This telephone screening is conducted with both the prospective participant and their prospective caregiver using screening questionnaires, and assesses the participant’s age (≥55 years), a physician diagnosis of memory impairment, and the principal exclusion criteria (history of stroke or other neurodegenerative or neurological disease, major psychiatric disorder, unstable cardiac disease, active cancer, seizures or epilepsy, implanted medical devices, scalp dermatological conditions, and other MRI or tACS contraindications), together with caregiver eligibility (age ≥21 years, computer and touchscreen proficiency, adequate vision, and availability to administer the home-based sessions). Eligible individuals are then invited to an in-person screening visit, during which inclusion and exclusion criteria are assessed. Written informed consent is obtained prior to participation in the study. Participants must also identify a caregiver or trained study staff member who will administer the home-based stimulation sessions.

#### 3.2.1 Inclusion criteria

Participants are eligible for inclusion if they are aged 55 years or older and have a clinical diagnosis of MCI based on a comprehensive clinical evaluation and standardized neuropsychological assessment. The neuropsychological examination covers language, visuospatial ability, executive function, and memory, and the diagnosis follows established clinical criteria for MCI, which require objective cognitive impairment with largely preserved functional independence and the absence of dementia. Consistent with the registered protocol (ClinicalTrials.gov: NCT05708001), MCI is established by expert clinical diagnosis and confirmed by the study physicians based on the participant’s cognitive evaluation and history, rather than by a single fixed psychometric threshold. Because the intervention targets the memory network, the study focuses on amnestic MCI, that is, participants with clear memory impairment on neuropsychological testing, who may also show impairment in other cognitive domains (single- or multiple-domain amnestic MCI). The MCI subtype is recorded at baseline. Eligible participants must be able to understand the study procedures, provide written informed consent, and be willing and able to comply with all study requirements. In addition, each participant must have a caregiver or accept a trained study staff member to administer the home-based stimulation sessions. Caregivers or stimulation administrators must be at least 21 years old, demonstrate sufficient proficiency with a computer or tablet, be willing to learn the stimulation procedures, not be pregnant, and be available to administer stimulation sessions throughout the intervention period.

#### 3.2.2 Exclusion criteria

**Figure 2:**
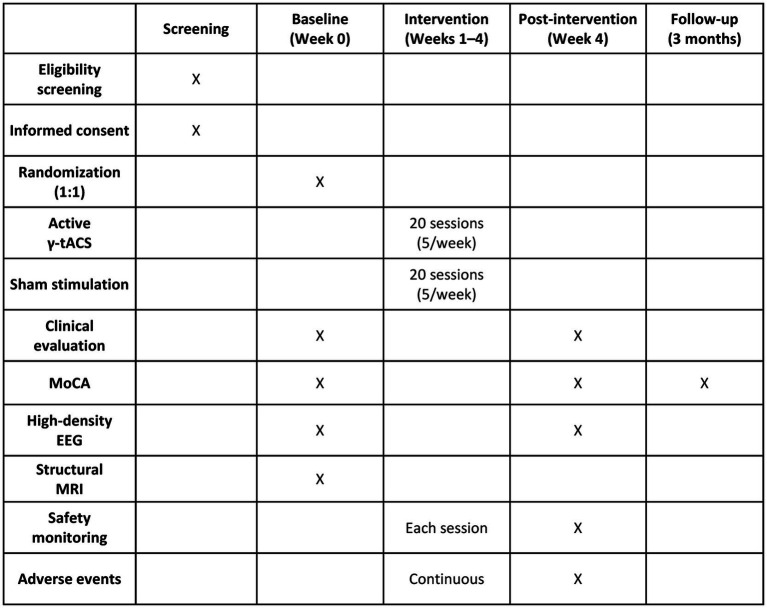
Schedule of enrolment, interventions, and assessments in the MemStim randomized controlled trial. Participants undergo eligibility screening and baseline assessments, including cognitive evaluation, high-density electroencephalography (hdEEG), and structural MRI. After randomization (1:1), participants receive either active gamma-frequency transcranial alternating current stimulation (γ-tACS) or sham stimulation during 20 home-based sessions over 4 weeks (five sessions per week). Cognitive and neurophysiological outcomes are assessed at baseline and after completion of the intervention. Cognitive assessment is also performed at the three-month follow-up visit. Safety monitoring and adverse events are recorded throughout the intervention period.

Participants are excluded from the study if they have a current psychiatric disorder such as schizophrenia, bipolar disorder, or major depressive disorder. Individuals with neurological disorders other than MCI, including Parkinson’s disease, multiple sclerosis, stroke, traumatic brain injury, or brain tumors, are also excluded. Additional exclusion criteria include a history of epilepsy or seizures, contraindications to MRI or non-invasive brain stimulation, and the presence of implanted electrical or metallic devices in the head or body. Participants are further excluded if they present with severe dermatological conditions affecting the scalp, unstable or severe medical conditions that could interfere with participation in the study, or an inability to provide informed consent. The study schedule of enrolment, interventions, and assessments is presented in Figure 2.

### 3.3 tACS parameters, home-based administration, and remote monitoring

Transcranial alternating current stimulation (tACS) is delivered using the Starstim SS32 (Neuroelectrics) device through surface Ag/AgCl electrodes placed in a neoprene cap following the international 10–20 EEG system. Individualized stimulation montages are generated using electric field modeling based on each participant’s structural MRI. Specifically, a personalized head model is reconstructed from each participant’s T1- and T2-weighted MRI scans and processed using SimNIBS (Thielscher et al., 2015; Windhoff et al., 2013) to optimize electrode configurations targeting nodes of the episodic memory network using the Stimweaver algorithm (Ruffini et al., 2014). Building on our prior work proposing personalized gamma-frequency tACS targeting the angular gyrus network to reduce memory deficits in AD (Bréchet et al., 2021b), the stimulation protocol uses a six-electrode montage designed to maximize the electric field in the target region while minimizing current spread to non-target areas. This individualized modeling follows the pipeline we validated in the present trial framework, in which MRI-based multi-electrode montages produced significantly stronger induced electric fields at the target than standardized, non-individualized montages in patients with MCI (Rochas et al., 2025); the optimization criterion is to maximize the normal component of the induced field at the left angular gyrus. Active stimulation consists of 40 Hz gamma-frequency tACS delivered for 20 min per session. The maximum injected current per electrode is 2 mA, with a maximum total current of 4 mA, producing an average electric field of approximately 0.25 V/m.

Current is ramped up over 30 s at the start and ramped down over 30 s at the end of each session to minimize discomfort. Sham stimulation is delivered using an ActiSham configuration optimized to each participant using the same algorithm as active stimulation, but with the target electric field set to zero, ensuring that the same electrode positions and number of electrodes are used in both conditions to guarantee operator blinding while effectively shunting current through the scalp without inducing meaningful cortical electric fields (Neri et al., 2020).

Participants receive 20 stimulation sessions over 4 weeks (five sessions per week). Stimulation sessions are conducted by trained caregivers or study personnel who undergo a structured training program prior to the intervention phase. Before and after each session, participants complete a standardized questionnaire to report any side effects. Remote monitoring is performed through the Neuroelectrics online portal, allowing research staff to supervise stimulation sessions in real time, including electrode impedance, stimulation parameters, and session progress. Session-level logs from this portal (electrode impedance, delivered current, stimulation duration, and completion) provide an objective record of stimulation fidelity and protocol compliance, and deviations from the prescribed schedule are documented. Adherence is summarized descriptively and reviewed at a planned interim analysis; the primary analysis follows the intention-to-treat principle, complemented by a per-protocol sensitivity analysis restricted to adherent participants. Participants and caregivers can communicate with study staff via videoconference or phone during stimulation sessions if needed.

### 3.4 Randomization and blinding

Participants are randomly assigned to either the active stimulation group or the sham stimulation group using a computer-generated randomization schedule. Allocation is concealed from both participants and the study personnel responsible for outcome assessment. The study follows a double-blind design in which neither the participants nor the investigators conducting the assessments are aware of the stimulation condition. The stimulation device is programmed to deliver either active or sham stimulation using predefined protocols that are indistinguishable to both participants and operators. To assess the integrity of blinding, at the end of the 4-week intervention, each participant and the tACS-administrator (caregiver) independently judge whether they believe the participant received active or sham stimulation and rate their certainty.

### 3.5 Outcome measures

#### 3.5.1 Safety and tolerability monitoring

Safety and tolerability of the intervention are assessed by monitoring adverse events, device deficiencies, and device-related side effects throughout the study. Participants complete standardized questionnaires before and after each stimulation session to report any discomfort or adverse effects. Common side effects of transcranial electrical stimulation include mild tingling, itching, or transient skin irritation at the electrode sites (Antal et al., 2017). All adverse events and device deficiencies are documented and monitored in accordance with ISO 14155 requirements. Adherence to the stimulation protocol, participant retention, and caregiver-reported acceptability are monitored as indicators of trial quality using a structured Study Evaluation questionnaire. Participants are instructed to report any discomfort or adverse effects during stimulation sessions, and remote monitoring allows the research team to supervise stimulation parameters and session progression in real time. A study physician reviews all adverse events and determines whether they are related to the stimulation procedure.

#### 3.5.2 Neuropsychological and behavioral assessments

Global cognitive function is assessed using the Montreal Cognitive Assessment (MoCA) (Nasreddine et al., 2005) at baseline, at the end of the intervention period, and at the three-month follow-up visit. The MoCA is validated and widely used in MCI, is sensitive to change, and has served as an outcome in prior gamma-frequency tACS trials in cognitive impairment (Cantoni et al., 2025; Benussi et al., 2022), enabling direct comparison of effect sizes. Because practice effects are expected to be comparable across the active and sham arms, the sham-controlled, parallel-arm design and the group × time interaction that constitutes the primary analysis inherently control for test–retest learning; the same applies to the repeated neuropsychological measures. To further mitigate practice effects, the three official alternate MoCA forms (versions 8.1, 8.2, and 8.3) are administered in counterbalanced order across the baseline, post-intervention, and follow-up visits (Costa et al., 2012), and alternate forms are used for the list-learning measures where available. The MoCA Memory Index Score additionally provides a memory-weighted readout.

In addition, a comprehensive neuropsychological battery is administered at baseline and post-intervention to characterize cognitive function across multiple domains. Global cognition is further assessed using the Mini-Mental State Examination (MMSE) and the Three Objects–Three Places (3O3P) test. Visuospatial and constructional ability is additionally assessed using the Clock Drawing Test. Episodic memory is evaluated using the Immediate, Free, and Cued Selective Reminding Tests (ISRT, FSRT, and CSRT), the Rey-Osterrieth Complex Figure test (copy and delayed recall), Logical Memory Story B (immediate and delayed), the Dubois Five-Word test, and the MoCA Memory Index Score. Working memory and attention are assessed using the Digit Span and Digit Symbol Coding subtests of the Wechsler Adult Intelligence Scale–Fourth Edition (WAIS-IV). Language is evaluated using semantic and phonemic verbal fluency tasks. Executive function is assessed using the Trail Making Test parts A and B (TMT-A, TMT-B). Mood symptoms are assessed using the Hospital Anxiety and Depression Scale (HADS).

Where available from the routine clinical diagnostic work-up, AD-relevant biomarker information is also recorded and summarized descriptively to characterize the biological profile of the enrolled cohort, including visual MRI rating scales (medial temporal atrophy [MTA], posterior atrophy [Koedam], and white-matter hyperintensities [Fazekas]), ApoE genotype, and amyloid, tau, and FDG PET in clinically indicated patients.

#### 3.5.3 MRI preprocessing and analyses

Structural MRI scans are used to generate individualized head models for electric field simulations. T1- and T2-weighted images with isotropic 1 mm resolution are processed using SimNIBS (Thielscher et al., 2015; Windhoff et al., 2013). Where a clinical MRI of sufficient quality obtained within the preceding 12 months is available, it may be used in place of a study-specific scan. The resulting head models allow estimation of the normal component of the electric field at the grey/white matter surfaces, enabling optimization of the injected currents across the electrode array to target the left angular gyrus.

#### 3.5.4 EEG recordings and analyses

We selected hdEEG rather than resting-state fMRI for three reasons. First, the intervention targets gamma-band (40 Hz) oscillatory activity, which tACS entrains directly; a time-resolved electrophysiological signal is therefore the appropriate mechanistic biomarker, whereas the BOLD signal cannot resolve gamma-frequency dynamics. Second, the structural MRI acquired for each participant already provides an individual head model for source-level EEG analysis, so network-level inference is available from the hdEEG data themselves. Third, an EEG-based readout suits the pragmatic, home-based design and limits participant burden in this older, cognitively impaired population.

High-density EEG (hdEEG) recordings are acquired at baseline and after completion of the intervention using a 257-channel system with Ag/AgCl sponge electrodes providing full scalp coverage. EEG signals are recorded at a sampling rate of 1,000 Hz and band-pass filtered between DC and 200 Hz, with the vertex electrode (Cz) used as the acquisition reference. Impedance levels are maintained below 30 kΩ. EEG preprocessing follows the procedures we have previously described and validated in Tarailis et al. (2025), including artifact rejection (removal of ocular and muscle components), interpolation of noisy channels, and re-referencing to the average reference.

Primary electrophysiological analyses focus on gamma-band power, which is quantified during a 5-min eyes-closed resting-state recording. EEG is also recorded during task-based conditions designed to engage memory-related neural networks, including a personalized autobiographical memory (ABM) task, in which participants judge whether photographs, i.e., personal images from their own life interleaved with neutral images, are self-related, probing self-referential autobiographical memory and engaging the angular-gyrus / hippocampal-cortical stimulation target [following Bréchet et al. (2021b)], and a recognition-memory control task requiring old/new judgments on neutral images.

hdEEG is projected into source space using each participant’s individual MRI-based head model and a distributed linear inverse solution [LORETA (Pascual-Marqui et al., 1994)], and the solution space is parcelled into regions of interest for the autobiographical memory network. Source-level gamma-band power is quantified in the angular gyrus and hippocampal target regions, together with EEG microstate temporal dynamics and theta–gamma cross-frequency coupling between the angular gyrus (gamma) and hippocampus (theta).

### 3.6 Statistical analysis

The primary outcome is the change in global cognitive performance, as measured by the Montreal Cognitive Assessment (MoCA), from baseline to post-intervention and follow-up. The secondary outcome is the change in gamma-band oscillatory activity measured with hdEEG from baseline to post-intervention. To evaluate the effects of the intervention, repeated-measures analyses of variance (ANOVA) are conducted using a 2 (group: active tACS, sham) × 3 (time: baseline, post-intervention, follow-up) mixed design for the primary outcome, and a 2 (group: active tACS, sham) × 2 (time: baseline, post-intervention) mixed design for the secondary outcome, reflecting the fact that hdEEG is not acquired at the follow-up visit. Analyses examine the main effects of group and time, as well as their interaction. Significant interaction effects are followed up with pairwise comparisons between baseline and post-intervention, and between baseline and follow-up, for each group separately.

Missing data are assumed to be missing at random; as a pre-specified complementary analysis, linear mixed-effects models (with fixed effects of group, time, and their interaction and a random intercept per participant) are fitted to use all available observations without listwise deletion, and their results are compared with those of the repeated-measures ANOVA. All analyses follow the intention-to-treat principle, and the extent and pattern of missing data are reported.

To account for inter-individual variability in delivered dose, the modeled electric-field intensity (V/m) at the target is extracted for each participant from the individualized head model, summarized descriptively across the cohort, and included as a participant-level covariate in exploratory dose–response analyses relating induced field strength to the cognitive and electrophysiological outcomes.

Sample size was estimated based on the primary outcome, change in MoCA score from baseline to post-intervention. Benussi et al. (2022) reported a large between-group effect of gamma-frequency tACS on episodic memory in early Alzheimer’s disease (Cohen’s d ≈ 1.0), with comparable effect magnitudes subsequently reported for home-based gamma-tACS in Alzheimer’s disease (Cantoni et al., 2025). Assuming a similarly large effect size (d = 1.0) on the primary outcome, a two-tailed independent-samples *t*-test with *α* = 0.05 and 80% power requires approximately 17 participants per arm. Allowing for a 15–20% attrition rate consistent with home-based, caregiver-administered neuromodulation trials in older adults with cognitive impairment, we set an enrolment target of 40 participants (20 per arm). The resulting sample size is expected to remain adequate to detect the pre-specified large effect size on the primary outcome.

All data collected during the study are stored on Research Electronic Data Capture (REDCap), a secure web-based application for building and managing clinical research databases (Harris et al., 2019; Harris et al., 2009). Statistical significance will be defined as *p* < 0.05.

## 4 Discussion

The MemStim study aims to investigate whether personalized, home-based gamma-frequency tACS can improve cognitive function in individuals with MCI. Given the limited treatment options currently available for early stages of neurodegenerative disease and the scarcity of evidence in MCI populations specifically, the development of scalable non-invasive neuromodulation strategies targeting neural network dysfunction represents a promising therapeutic direction. By integrating personalized MRI-guided stimulation targeting, electrophysiological biomarkers, and remotely supervised home-based delivery, the MemStim study represents one of the first randomized trials to evaluate gamma-frequency tACS specifically in MCI, a population in which early intervention may have the greatest potential to alter disease trajectories.

The present protocol builds on a growing tradition of network-guided non-invasive brain stimulation in dementia, in which functional imaging is used to define disease-relevant network targets rather than isolated anatomical sites (Pievani et al., 2016). Within this framework, stimulation of default-mode-network hubs has produced clinical and neurophysiological effects in Alzheimer’s disease; for example, repetitive TMS of the precuneus (Koch et al., 2022) and multifocal transcranial electrical stimulation have been used to modulate the default-mode and salience networks in Alzheimer’s disease and frontotemporal dementia (Pini et al., 2022).

The left angular gyrus targeted here is a lateral-parietal hub of the hippocampal-cortical autobiographical memory network and the posterior default mode network, consistent with our prior work implicating the angular gyrus in episodic autobiographical memory (Bréchet et al., 2019; Bréchet et al., 2018). The present protocol extends this network-targeting logic by combining MRI-individualized, hdEEG-informed multifocal montages with frequency-specific entrainment: rather than modulating network connectivity per se, we aim to entrain gamma-band activity at the angular-gyrus node of the memory network, coupling network-level targeting with oscillation-specific modulation.

The present study also contributes to the growing body of research investigating gamma-frequency modulation as a therapeutic strategy in AD. Experimental studies have shown that externally driven gamma oscillations may influence pathological processes associated with neurodegeneration, including amyloid deposition and microglial activation (Iaccarino et al., 2016). While the clinical relevance of these mechanisms in humans remains an active area of investigation, emerging neuromodulation studies suggest that restoring gamma-band synchronization may be a promising avenue for improving cognitive function (De Paolis et al., 2024; Koch et al., 2024). The inclusion of hdEEG as a secondary outcome measure will allow direct examination of whether the stimulation protocol modulates gamma-band activity in targeted memory networks, providing a neurophysiological basis for interpreting any observed cognitive effects.

A central contribution of the MemStim study is the direct examination of whether gamma-frequency tACS can modulate neural dynamics in memory-related neural circuits in individuals with MCI. Unlike previous studies that have predominantly focused on AD populations (Benussi et al., 2022; Cantoni et al., 2025; Tang et al., 2024) or evaluated stimulation in laboratory settings without individualized MRI-based targeting (Mirjalili et al., 2025), the MemStim study uniquely combines personalized electric-field modeling, home-based delivery, and multimodal outcome assessment in an MCI population. If gamma-band power in targeted memory networks increases following active stimulation relative to sham stimulation and is associated with improvements in MoCA scores, this would provide important mechanistic evidence supporting the use of frequency-specific neuromodulation as a circuit-based intervention for early cognitive decline (Koch et al., 2024). Conversely, dissociations between electrophysiological and cognitive outcomes would offer valuable insights into the conditions under which oscillatory modulation translates into functional benefits.

In addition to evaluating cognitive outcomes, the study incorporates high-density EEG to assess changes in neural oscillatory activity. The inclusion of neurophysiological biomarkers may provide valuable insights into the mechanisms underlying potential cognitive improvements and help determine whether behavioral effects are accompanied by modulation of neural activity. Such multimodal approaches, combining behavioral and neurophysiological measures, are increasingly recognized as important for advancing neuromodulation research and improving the reproducibility of stimulation effects (Vosskuhl et al., 2018; Koch et al., 2024). Beyond the present trial, neurophysiological biomarkers derived from hdEEG may help identify individuals most likely to respond to gamma-frequency neuromodulation, and future studies may investigate whether longer or repeated stimulation protocols produce more sustained cognitive benefits (De Paolis et al., 2024; Benussi et al., 2022).

## 5 Limitations

Several limitations of the study design should be acknowledged. First, the sample size, while powered to detect moderate-to-large effects reported in prior gamma-frequency tACS trials, may be underpowered for smaller effects; effect-size estimates from this trial will inform the design of future, larger studies. Second, although home-based stimulation increases accessibility, it introduces potential variability in session administration despite standardized caregiver training and real-time remote supervision. Third, inferring large-scale network dynamics from scalp EEG has lower spatial resolution than fMRI, particularly for deep generators such as the hippocampus; we mitigate this by using individual MRI-based head models and a source-imaging approach previously validated for subcortical activity (Seeber et al., 2019), and we interpret source-level findings as convergent electrophysiological evidence rather than a direct measure of hemodynamic connectivity. The cohort is defined clinically rather than by molecular biomarkers, which are not systematically acquired as part of the protocol; the enrolled sample is therefore likely biologically heterogeneous and may include participants whose MCI is not due to Alzheimer pathology, potentially attenuating intervention effects and constraining mechanistic interpretation. A clinically defined population nonetheless reflects the intended real-world, scalable deployment context of a home-based intervention, and the effect-size estimates from this trial will inform biomarker-stratified confirmatory designs. Finally, while the present trial focuses on gamma-frequency stimulation based on prior evidence linking gamma oscillations to memory processes, the optimal stimulation parameters for neuromodulation in MCI remain an area of ongoing investigation.

## 6 Conclusion

The MemStim study represents an important step toward developing personalized, scalable neuromodulation interventions for early-stage cognitive decline. By integrating individualized stimulation targeting, remote monitoring technologies, and electrophysiological biomarkers, the present trial aims to advance our understanding of how modulation of neural oscillations may influence cognitive function in individuals with MCI. The inclusion of hdEEG as a secondary outcome measure further enables the identification of neurophysiological markers that may help stratify individuals most likely to benefit from gamma-frequency neuromodulation, informing future patient selection strategies. If successful, the MemStim study may provide important evidence supporting personalized neuromodulation strategies targeting neural network dysfunction in early AD. Moreover, the combination of individualized stimulation targeting and remotely supervised home-based delivery, demonstrated to be feasible and acceptable in prior work (Bréchet et al., 2021b; Bouhour et al., 2026), could represent a scalable therapeutic approach that reaches larger populations at risk of cognitive decline.
